# Strengths and weaknesses of the MABC-2 as a diagnostic tool for developmental coordination disorder: An online survey of occupational therapists and physiotherapists

**DOI:** 10.1371/journal.pone.0286751

**Published:** 2023-06-02

**Authors:** Kathryn J. Hadwin, Greg Wood, Sally Payne, Christopher Mackintosh, Johnny V. V. Parr

**Affiliations:** 1 Allied Health Professions Research Unit, University of Central Lancashire, Preston, United Kingdom; 2 Department of Sport and Exercise Sciences, Manchester Metropolitan University Institute of Sport, Manchester, United Kingdom; 3 Royal College of Occupational Therapists, London, United Kingdom; The University of Sydney, AUSTRALIA

## Abstract

The Movement Assessment Battery for Children-2 (MABC-2) is the most widely used instrument for aiding the diagnosis of developmental coordination disorder (DCD). Despite being shown to have strong validity and reliability, it has received criticism for aspects of its scoring system, the lack of formal training, and its susceptibility to overlook higher functioning DCD children. To aid the development of future diagnostic tools and/or iterations of the MABC-2, the present study attempted to draw upon the experience of key stakeholders and determine the strengths and weaknesses of the MABC-2. Using a short online questionnaire, occupational therapists (*n* = 14) and physiotherapists (*n* = 3) with experience using the MABC-2 for DCD diagnosis completed a series of Likert scale and free-text questions. Braun and Clarke’s six-phase process to thematic analyses was used to identify main themes obtained across quantitative and qualitative data. Results indicate that whilst the MABC-2 is easy to administer and interpret, the scores can misrepresent true motor difficulties due to (a) daily variations in mental and physical state, (b) the reliance on non-functional tasks, (c) negative interference from parents, (d) changes in motor competency due to practice, and (e) a lack of formal examiner training to ensure the test is effectively lead. Further work is needed to more reliably determine how perceptions of the MABC-2 might vary across levels of expertise, profession, and cultural differences.

## 1. Introduction

Developmental coordination disorder (DCD) is a neurodevelopmental disorder characterised by difficulties in general motor skill learning and execution, resulting in marked interference with activities of daily living [1]. Estimates suggest that around 5 to 6% of school children live with DCD [1] and that, without proper diagnosis and interventions, three quarters of children with DCD will continue to have difficulty into adulthood, negatively impacting employability [2].

Despite the prevalence of DCD, its aetiology is still unknown, with some suggesting that its heterogeneity makes pinpointing an exact cause extremely difficult [3]. Whilst DCD appears to manifest on the neurophysiological level [4], its diagnosis is highly determined on a behavioural level and assessed via motor performance. Typically, children are diagnosed or identified between the ages of 6 to 12 years of age [5] and must undergo a diagnostic process that meets the DSM-5 criteria [1]. Central to this process is a physical assessment, the most common of which is the Movement Assessment Battery test for Children 2 [6] (MABC-2). The MABC-2 is the most widely used instrument for diagnosing children up to the age of 16 with movement disorders [7]. The MABC-2 contains eight tasks that challenge static balance, dynamic balance, manual dexterity, and ball skills, each of which are adapted across three age ranges (3–6 years, 7–10 years, 11–16 years). Performance on each task is assessed primarily through task completion times (using a stopwatch) and/or task success. Upon completion, the total standardised score categorises each child into a percentile that identifies each child as ‘*not at risk*’, ‘*at risk*’, or ‘*likely to have motor coordination impairment*’. The MABC-2 also includes a questionnaire-based checklist (i.e., the MABC-2 checklist) designed for teachers, parents, and therapists to provide feedback on the ability of the child to perform motor skills in natural environments such as school, family, and community settings.

Several key advantages of the MABC-2 have been documented. For example, both the validity and reliability of the test for discriminating between specific motor abilities have received extensive support [8, 9]. It has also been praised for its international availability, and its cross-cultural validity [10, 11]. Finally, the test has also been praised for its easy administration, which facilitates large sample screening over a short period [12]. However, there are several possible limitations of the MABC-2 that could reduce its sensitivity and suitability for the diagnosis of DCD. For example, the overall test score is based on the combined scores on each sub-task category. Consequently, poor performance on one sub-category (e.g., manual dexterity) could be neutralised by excellent performance on another sub-category (e.g., balance) [5]. French et al. [13] also suggested that the scoring system of the MABC-2 suffers from a “ceiling effect” as many of the tasks require only two trials, with only the best performed trial contributing to the final score. As a result, only an individual’s maximal performance is considered, overlooking any variance observed (e.g., several extremely poor trials) and thus overestimating motor performance. Additionally, individuals who perform well progress through the test more rapidly and engage in fewer trials overall. Poorer performing individuals will therefore take longer to complete the assessment, and as a result become tired and unmotivated [13].

The MABC-2 is also typically carried out by a wide variety of professionals, including occupational therapists, physiotherapists, psychologists, researchers, and educational professionals [14]. Consequently, the degree of experience and training an examiner has in identifying clinical movement disorders is also likely to vary. Given the MABC-2 includes a subjective assessment component, it appears crucial that MABC-2 examiners have sufficient training and knowledge about movement disorders to prevent a child from being inaccurately screened and thus prevented from being sent to a paediatrician. Finally, the MABC-2 also possibly overlooks the trainability of a child with DCD. To elaborate, extensive literature has shown that children with DCD can show marked improvements in their motor skills following targeted interventions [15]. Scores on the MABC-2 may, therefore, underestimate the motor difficulties of children who have had a greater level of support prior to examination.

Despite the popularity of the MABC-2, its critical evaluation is required to aid the development of future diagnostic tools and/or iterations. To achieve this, it is important to engage with key stakeholders who hold extensive practical and professional experience. Therefore, the aim of this study was to understand the perception of key stakeholders (i.e., occupational therapists, physiotherapists) on the strengths and weaknesses of the MABC-2 for the diagnosis of DCD in children, using an online survey.

## 2. Methods

### 2.1 Approach to the problem

A cross-sectional anonymous online survey was conducted to evaluate the perceptions of healthcare professionals on the utility of the MABC-2 for diagnosing DCD. The study was conducted as part of a postgraduate degree in physiotherapy. Ethical approval was received from the University of Central Lancashire and consent was obtained from each participant before completing the questionnaire. Permission from NHS trusts was accepted from the head of research and development.

### 2.2 Survey design and development

Participants completed an anonymous and non-validated online questionnaire that was developed for this project, with all data collected through Qualtrics Research Suite (Provo, UT). The questionnaire consisted of three sections: (a) study information sheet and informed consent, (b) demographics and information regarding the experience with and frequency of using the MABC-2 for DCD diagnosis, and (c) a combination of both Likert scale and free text questions that allowed respondents to share both quantitative and qualitative perceptions of their experience. The Likert scale questions served to provide insights to the magnitude and frequency of perceptions. By contrast, the free text questions gave participants the flexibility to discuss relevant topics that build upon the initial question. This provides participants with greater control, which can lead to the generation of more meaningful responses and a deeper understanding of the topic [16]. The content of the questions was designed to build upon previously discussed strengths and weaknesses of the MABC-2 across the literature base. The individual questions were then refined using the feedback of one senior occupational therapist and one senior physiotherapist, both of whom have substantial experience implementing the MABC-2 for the diagnosis of DCD. As such, participants were asked several questions broadly pertaining to the strengths and weaknesses of the MABC-2 for DCD diagnosis, and how the MABC-2 could be improved for this purpose. Based on previous research, we further explored issues regarding the effectiveness of the scoring system, the likelihood of children being able to improve their MABC-2 scores, and whether enough training had been received to effectively administer the MABC-2. Following discussions with the senior healthcare workers, it was then decided to additionally explore how the examiner and parents might influence performance on the MABC-2 independent of the child’s motor difficulties. A copy of the final questionnaire can be found in S1 File.

### 2.3 Participants and recruitment

A total of 25 respondents started the questionnaire, and 17 respondents fully completed the questionnaire (68% completion rate). Fourteen of the respondents were occupational therapists (OT), whilst three were physiotherapists (PT). Twelve respondents reported to use the MABC-2 for the diagnosis of DCD between one and seven times per month, three respondents reported to use the MABC-2 more than eight times per month, and two reported to no longer use the MABC-2 (Table 1). Participants were recruited through a child development centre in the Northwest of England and via social media promotion. Participants were required to be paediatric practitioners and have experience using the MABC-2 for DCD diagnosis. Participants were excluded if they were not a practising paediatric PT or OT or had no formal experience using the MABC-2 for DCD diagnosis. Data collection took place between January and June of 2022.

**Table 1:** Demographic information of respondents, indicating their ID, their occupation, and how many times per month they currently use the MABC-2 for DCD diagnosis in children.

Respondent ID	Occupation	MABC-2 assessments per month (currently)
PT1	Physiotherapist	0
PT2	Physiotherapist	1–7
PT3	Physiotherapist	22
OT1	Occupational therapist	1–7
OT2	Occupational therapist	1–7
OT3	Occupational therapist	1–7
OT4	Occupational therapist	1–7
OT5	Occupational therapist	1–7
OT6	Occupational therapist	1–7
OT7	Occupational therapist	0
OT8	Occupational therapist	1–7
OT9	Occupational therapist	1–7
OT10	Occupational therapist	1–7
OT11	Occupational therapist	1–7
OT12	Occupational therapist	1–7
OT13	Occupational therapist	8–14
OT14	Occupational therapist	8–14

### 2.4 Data analysis

Quantitative data were summarised via frequencies (*n*) and median values. For qualitative data, Clarke and Braun’s six-phase process of thematic analysis [17] was used to identify patterns or themes within the data because this approach provided the researcher with a descriptive account of the construct under study. Thematic analysis provides a comprehensive story of the interpretations and experiences of the individuals under study [16, 17]. This process involved the researcher first familiarising themselves with the free-text responses by reading them several times to identify broad statements of interest. The researcher then engaged in complete coding to identify larger patterns or themes within the data. Finally, the themes were named and defined to capture the overarching essence of each individual theme. The lead author initially conducted the thematic analysis and subsequently shared and discussed it with two other authors, one of whom is experienced in this analytical approach.

## 3. Results and discussion

### 3.1 Quantitative data

Respondents generally reported the usefulness (median = 6/10) and functionality (median = 5/10) of the MABC-2 for diagnosing DCD as moderate. The effectiveness of the scoring system scored slightly higher, achieving a median score of 7 on a scale of 1 (not effective at all) to 10 (extremely effective). There was a strong consensus that children can increase their MABC-2 score with practice, with 16 respondents answering either “*Probably yes*” (*n* = 11) or “*Definitely yes*” (*n* = 5). Most respondents also felt as though the examiner can have some influence on MABC-2 scores, with 13 participants answering either “*Probably yes*” (*n* = 7) or “*Definitely yes*” (*n* = 6). Most participants also indicated that the parents watching might have *some* impact on the child’s performance (*n* = 15), answering either “*Might or might not*” (*n* = 5) or “*Probably yes*” (*n* = 5), or “*Definitely yes*” (*n* = 5). Finally, when asked whether they had received enough training to lead the MABC-2, most participants answered, “*Probably yes*” (*n* = 7), with fewer answering either “*Probably not*” (*n* = 3) or “*Might or might not*” (*n* = 4), or “*Definitely yes*” (*n* = 3).

### 3.2 Qualitative data

#### Sub-themes within each free-text question

When asked to reflect on the benefits of the MABC-2 for DCD diagnosis, the most common themes were the ease of administration (*n* = 6), its ability to simply identify each child’s motor difficulties (*n* = 4), and the extent to which it can aid formal diagnosis (*n* = 4). When asked to reflect on the challenges of using the MABC-2 for DCD diagnosis, the most common theme was that scores are often not reflective of motor difficulties due to its non-holistic approach (*n* = 11). Another common theme was that the tasks included in the MABC-2 were not functional and thus not representative of the tasks children face daily (*n* = 6). When asked how the MABC-2 can be improved, the common themes included the inclusion of functional tasks (*n* = 8), improvements to the scoring system (n = 3), a wider range of age intervals (*n* = 3), and improvements to the administrative guidance (*n* = 3). When specifically asked about the effectiveness of the scoring system, common themes indicated that the score is often not reflective of motor difficulties (*n* = 5), that subjective interpretation could influence results (*n* = 2), and that the scoring system is easy to understand (*n* = 4). When asked whether children can improve their MABC-2 score, a common theme indicated that children can improve their scores after targeted interventions (*n* = 10). However, another common theme indicated that scores could be improved, but as the tasks depend on splinter skills, their development would be redundant for activities of daily living (*n* = 4). When asked to elaborate on how the examiner can influence performance on the MABC-2, a common theme related to the lack of clear administrative guidance on how to instruct and interact with each child (*n* = 15, e.g., demonstrating, over-explaining, reassuring, motivating). Another common theme related to the subjective interpretation of performance, which can vary between and within examiners (*n* = 4). When asked to elaborate on how the parents can influence a child’s performance, a common theme suggested that parents can often negatively interfere with a child’s performance (*n* = 15), whilst another theme indicated that parents can provide positive reassurance (*n* = 2). Finally, when asked to elaborate on whether they had received enough training, a common theme indicated that many respondents had received no formal training (*n* = 11), and that formal training would be beneficial (*n* = 4).

#### Main themes

Following the analysis, two main themes were identified and were labelled “Strengths of the MABC-2” and “Weaknesses, concerns and challenges of the MABC-2”. The following section provides descriptions and supporting quotations from the respondents to illustrate the themes more broadly within the context of the literature base.

### 3.3 Strengths of the MABC-2

This theme specifically referred to the strengths of the MABC-2 as a diagnostic tool for children with DCD. Despite only moderate levels of satisfaction with the MABC-2, several respondents (*n* = 6) praised the ease of administration. For example, one respondent reported that the test is "*Quick*, *easy to set up and score…*” [*OT9*]. The ease of administration has been praised previously as it facilitates large sample screening over a short period of time [12], a factor clearly beneficial to a stretched NHS system [18]. However, it should be noted that time constraints are consistently highlighted as a barrier to health promotion among physiotherapists [19], who report that increased demands to work quickly comes at the cost of decreased quality in care—conflicting their professional standards and ethos [20]. Thus, whilst a time-efficient approach may be necessary it may not promote optimal assessment standards.

The simplicity of the scoring system was also praised by respondents, as it allows the child’s difficulties and test outcomes to be clearly defined:

“*I can see specifically the areas they struggle and can begin to target my interventions accordingly*”

[*OT2*]

“*The information given is helpful for explaining the child*’*s difficulties to parents*, *school staff and members of the MDT*”.

[*PT1*]

These findings align with subjective reports of primary school teachers, who also described the MABC-2 as easy to complete, analyse and interpret [21].

### 3.4 Weaknesses, concerns and challenges of the MABC-2

This theme specifically referred to the weaknesses, concerns and challenges of the MABC-2 as a diagnostic tool for children with DCD. From this theme, several sub-themes were identified: Score is not reflective of observed motor difficulties, The role of the distracting parent, Tasks lack functional relevance, Children can improve the score with practice, The role of the examiner.

#### Score is not always reflective of motor difficulties

Despite the potential benefits of a test that is simple to administer and interpret, many respondents highlighted some fundamental issues that may question its validity for the diagnosis of DCD. Many respondents felt that the achieved MABC-2 score can fail to reflect the observed and subjectively reported motor difficulties. For example, the non-holistic approach of the MABC-2 was thought to overlook daily variations in a child’s physical and mental state:

“*I feel that a child’s performance is dependent on many factors on the day*. *For example*, *their attention and concentration*, *their emotional wellbeing*, *their fatigue and their change in routine as they will have often missed school…*”.

[*OT2*]

This response aligns with evidence that individuals with DCD are highly susceptible to cognitive [22] and physical [23] fatigue. There is also a high prevalence of co-occurrence between DCD and attentional deficit hyperactivity disorder (ADHD), with as many as 50% of children with ADHD being diagnosed with DCD [24]. This raises the possibility that the outcome of assessment might be influenced by daily variations in a child’s hyperactivity-impulsivity and/or inattention during testing—a factor that is of particular concern if only conducted on a single occasion.

It was also highlighted that scores on the MABC-2 fail to capture the quality of movement:

“…*The quality of movement is so important and does not get taken into account at all…*”.

[*PT2*]

Indeed, research has shown that children with DCD can display fundamental differences in their movement patterns (e.g., greater variability in kinematics) in tasks such as walking [25] and stair climbing [26], despite no differences in task failures (i.e., trips and falls). Interestingly, it has been suggested that increased coordinative variability in children with DCD may reflect individualised coordination solutions depending on the specific constraints placed upon their neuromuscular system [27]. Placing emphasis on crude outcome measures of task performance may therefore overlook the possibility that performance outcomes were achieved with fundamentally greater coordinative variability, which may provide a more subtle indication that motor difficulties are present.

#### The role of the distracting parent

Perhaps surprisingly, most respondents indicated that parents have a negative impact upon the MABC-2 assessment. Whilst the presence of a parent or guardian generally holds emotional and ethical value, some respondents indicated that their enthusiasm to aid their child can interfere with the child’s performance:

“*…Parents may invertedly put their child off or may make them perform better than they usually would*”

[*OT7*]

“*…Parents can give clues*, *even if instructed not to*”

[*OT9*]

“*…Some children look to their parent for reassurance which sometimes seems to interrupt the child*’*s performance*”

[*OT14*]

These findings indicate that parents who intend to support the MABC-2 process might detract from the quality of assessment by altering the challenge of the task. For example, providing additional instructions to guide performance might give a child a performance advantage over a child who does not receive such support. By contrast, if parental support is distracting, it could unintentionally impair performance by drawing attention away from the task itself. Interestingly, several respondents also reported that parents can negatively impact the assessment by adding a degree of performance pressure:

“*Some children feel under pressure to please parents–whether this be by achieving a really good score or a really low score in order to achieve a referral to community paediatrics for diagnosis*”

[*PT1*]

“*…I also know parents get frustrated or emotional that the child cannot complete a simple task…*”

[*OT2*]

“*Sometimes parents encourage children to hurry up or stay focused…parents get emotional which will impact the child*’*s success*”

[*OT6*]

The possibility that parents can increase performance pressure is of particular concern given that pressure induced anxiety can directly influence the way we move [28]. Indeed, there is evidence that individuals with DCD experience higher levels of anxiety [29], and that motor control is related to task-specific anxiety in children with DCD [26]. Performance pressure from a parent could therefore impede some aspects of performance during the MABC-2 or simultaneously facilitate compensation in ways that ensure safe and effective movement completion [28]. Further work is needed to elucidate the influence of parental pressure on child performance during the MABC-2 so that recommendations for best practice can be developed.

#### MABC-2 tasks lack functional relevance

When asked about the challenges of using the MABC-2 and how the tool can be improved, a common concern was that tasks generally lack functional relevance and are limited in their variation and complexity. For example:

“*It does not give you a true diagnosis and you need to consider other elements such as their routines*, *adverse childhood events*, *opportunities they have*, *scores don*’*t tell a picture or give an accurate diagnosis*“

[*OT8*]

Indeed, criterion two of the DSM-5 states that motor difficulties must significantly and persistently interfere with activities of daily living or academic achievement. Another respondent suggested it would be more advantageous to:

“…*base it on observational assessments seen within a child*’*s real life context (e*.*g*., *handwriting*, *PE*, *cycling)*”.

[*OT7*]

It could be argued that the isolated nature of each MABC-2 task lacks contextual complexity and thus fails to expose each child to aspects of executive functioning required for everyday life (e.g., working memory, inhibitory control, and cognitive flexibility). For example, children will often be faced with performing these tasks whilst holding conversations, monitoring aspects of the environment, or whilst performing a secondary motor task (e.g., whilst walking). Despite growing evidence that executive function deficits are at the heart of the difficulties faced by individuals with DCD in these dual-task environments [30], it is perhaps surprising that no components of the MABC-2 are designed to expose them. Although the MABC-2 checklist is commonly used to subjectively assess motor difficulties in everyday scenarios, concerns have been raised regarding the tool’s validity, sensitivity, and inter-rater reliability [31]. To obtain more comprehensive and accurate insights into these difficulties, it might be necessary to include additional functional and complex motor tasks as part of the physical assessment. Such tasks could provide a more direct and complementary evaluation of the child’s motor ability d help identify areas that require further attention or intervention.

#### Children can improve their scores with practice

Most respondents agreed that children can display improvements in the MABC-2 scores with practice. For example:

“*All children can improve a skill practising the individual activities*”

[*OT4*]

“*We know that practice helps children and children with DCD can receive strategies to work on their goals*. *This leads to an improvement in functional skills*”.

[*OT2*]

These perceptions align with empirical evidence that children with DCD can improve fundamental motor skills, such as throwing, catching, and balance. Whilst these findings may not be immediately surprising, it highlights the limitations of relying on motor skills for the assessment of DCD. For example, it is possible that opportunities and encouragement to engage in motor skills throughout development could enhance motor proficiency and thus mask motor difficulties in the simpler environment imposed by the MABC-2.

#### The role of the examiner

Most respondents indicated that the examiners can, to some extent, influence the child’s performance independent of their motor difficulties. Many respondents suggested that the lack of clear administrative guidance could lead to within and between examiner differences in instructional and motivational styles (e.g., demonstrating, over-explaining, reassuring). For example:

“…*even though we have a manual*, *there is no script and we have been known as a team to have different approaches to delivering assessment and communicating with a child*”

[*OT2*]

“*Sometimes it is hard not to give extra instructions*. *Also deciding on a fail can be quite subjective*”.

[*OT1*]

The impact of differing styles was reinforced by those who stated:

“Y*our demeanour*, *approach*, *explanation and demonstration will impact performance*”

[*OT13*]

“*Children with DCD often have low self-esteem*. *A supportive examiner can ensure children demonstrate their optimum level of performance*”.

[PH1]

“…*the manual is very clear in terms of instructions for delivering and scoring and interpreting*, *but I think implementing requires good child centred communication skills…which can vary between therapists*”.

[*OT14*]

Given the clear impact that examiner style can have, it is interesting to note that most respondents reported having never received any formal training:

“*I learned on the job (I have many years of experience now*!*)*. *I have shared my experience with other members of staff*, *but I’m not aware of any formal training people can access to help them administer and*, *more importantly*, *interpret findings*. *This is a gap*.”

[*OT3*]

“*I didn*’*t have any training it has only been through experience that I have developed my own skills and knowledge*”

[*OT8*]

It is therefore perhaps unsurprising that styles appear to vary across examiners. The lack of training could also be problematic as the MABC-2 includes a subjective assessment component that requires examiners to have sufficient training and knowledge about movement disorders. Indeed, it has been shown that a lack of training can cause deficits in the ability of PE teachers to assess fundamental movement skills and lower teacher confidence in delivering assessments [32]. Specialist, professional training may therefore be required to overcome inter- and intra-variability in examination styles and improve the robustness of assessment outcomes. That said, it should again be reinforced that the validity and reliability of the MABC-2 for discriminating between specific motor abilities has received extensive support [8, 9]. The disparity between these findings and the perceptions of stakeholders identified from our study is therefore unclear and should be addressed in future research.

#### Study limitations

Several limitations should be acknowledged from the present study. For example, we relied upon a non-validated questionnaire to provide preliminary and exploratory insights to how physiotherapists and occupational therapists perceive the MABC-2. Whilst the content of our questionnaire was developed with the guidance of a senior occupational therapist and a senior physiotherapist, technical expertise on the structure of the questionnaire was not obtained. The use of a non-validated questionnaire in this manner could therefore question the validity and reliability of our findings, and lead to potential biases and inaccuracies in our data. There are also serious limitations in utilising an anonymous online questionnaire. For example, we cannot guarantee that participants met the inclusion criteria or that participants only answered once. We also limited the depth of personal information we gathered from participants such as their experience (band of practice), gender, or country of residence. Our findings should, therefore, be generalised with caution until future research attempts to tease out these factors. That said, it should be recognised that there are many advantages of adopting online questionnaires that prioritise anonymity. For example, Braun et al. [33] indicate that anonymity can encourage participants to be more honest and open in their responses and can reduce the tendency for participants to provide responses that they believe are socially desirable rather than their true beliefs (i.e., social desirability bias). This can be particularly important when researching potentially sensitive topics and is well suited to preliminary investigations like ours. Finally, our sample was heavily skewed towards occupational therapists, questioning the reliability of generalising our findings to physiotherapists.

## 4. Conclusion

The findings of the present study highlight several perceived weaknesses of the MABC-2 for diagnosing DCD. It appears that key stakeholders feel MABC-2 performance can be influenced by several factors unrelated to motor difficulties. Whilst many of these factors might be common to most tests of this nature, future diagnostic tools and/or iterations of the MABC-2 could attempt to account for possible alterations in physical and mental fatigue, include a wider range of tasks that are more representative of daily life, and ensure examiners receive sufficient training to better standardise how both the examiner and parents might independently influence motor performance. If feasible, more objective measures of movement quality would provide an interesting way to support DCD diagnosis and even validate the MABC-2 across and within examiners.

## Supporting information

S1 File (PDF)
