# Supplementary material for: Strengths and weaknesses of the MABC-2 as a diagnostic tool for developmental coordination disorder: An online survey of occupational therapists and physiotherapists
Source: PLoS One. 2023 Jun 2;18(6):e0286751. doi: 10.1371/journal.pone.0286751 (PMC10237484; doi:10.1371/journal.pone.0286751)
Supplement: S1 File — (PDF) [file pone.0286751.s001.pdf]

MSc project

Start of Block: Default Question Block

Q1 The perceived usefulness and potential pitfalls of the Movement Assessment Battery for Children 2 (MABC-2) as a diagnostic assessment tool for developmental coordination disorder.

Research ethics approval number: HEALTH 0270

Dear potential participant,

You are being invited to take part in research to investigate “The perceived usefulness and potential pitfalls of the Movement Assessment Battery for Children 2 as a diagnostic assessment tool for developmental coordination disorder.”

This will involve the completion of an electronic questionnaire. The questionnaire should take you no longer than 10 minutes.

Prior to starting the questionnaire, please familiarise yourself with the patient information sheet available here: (https://tinyurl.com/yh4d4sh5)

Once you complete and submit the questionnaire you cannot withdraw your answers as all data will be anonymised upon submission.

If you have any questions or concerns, please contact:
Student Investigator, Kathryn Hadwin, khadwin@uclan.ac.uk

Thank you for reading this and taking part in this research

Q3 I confirm that I have read and have understood the information sheet for the above study.

I have had the opportunity to consider the information, ask questions and have had these answered satisfactorily.

I understand that I can stop completing the questionnaire at any time, but once submitted I cannot withdraw as responses are immediately anonymised.

I understand that the information I provide will be held securely and in line with data protection requirements at the University of Central Lancashire.

○ I consent (1)

End of Block: Default Question Block

Start of Block: Block 1

Q1 What is your profession?

○ Physiotherapy (1)

○ Occupational therapy (2)

○ Other (please specify) (3) ____

Q2 In a typical month, how many times do you use the MABC-2?

○ 0 (1)

○ 1-7 (2)

○ 8-14 (3)

○ 15-21 (4)

○ 22+ (5)

Q3 On a scale of 1-10 how useful do you think the MABC-2 is for diagnosing DCD?

	Not at all useful									Very useful	
	0	1	2	3	4	5	6	7	8	9	10
1 ()											

Q4 From your experience what are the benefits of the MABC-2 for diagnosing DCD?

____

Q5 In your experience, what are the challenges of using the MABC-2 for diagnosing DCD?

____

Q6 On a scale of 1-10 how functional do you perceive the MABC-2 to be for DCD diagnosis?

	Not functional at all									Very functional	
	0	1	2	3	4	5	6	7	8	9	10

1 ()	

Q7 How do you think the MABC-2 can be improved?

____

Q8 How effective do you think the MABC-2's scoring system is?

	Very ineffective						Very effective				
	0	1	2	3	4	5	6	7	8	9	10
1 ()											

Q9 Please explain your answer (Q8)

____

Q10 Do you think children with DCD can increase their MABC-2 score with practice?

- ○ Definitely not (1)
- ○ Probably not (2)
- ○ Might or might not (3)
- ○ Probably yes (4)
- ○ Definitely yes (5)

Q11 Please explain your answer (Q10)

____

Q12 Do you think you, as the examiner, can have an impact on the child's performance during the MABC-2 test?

- ○ Definitely not (1)
- ○ Probably not (2)
- ○ Might or might not (3)
- ○ Probably yes (4)
- ○ Definitely yes (5)

Q13 Please explain your answer (Q12)

____

Q14 Do you think the parents watching can have an impact on the child's performance during the MABC-2 test?

- ○ Definitely not (1)
- ○ Probably not (2)
- ○ Might or might not (3)
- ○ Probably yes (4)
- ○ Definitely yes (5)

Q15 Please explain your answer (Q14)

____

Q16 Do you feel you have had enough training to effectively lead the MABC-2?

- ◯ Definitely not (1)
- ◯ Probably not (2)
- ◯ Might or might not (3)
- ◯ Probably yes (4)
- ◯ Definitely yes (5)

Q22 Please explain your answer (Q16)

____

End of Block: Block 1
